# The relationship between smoking frequency and life satisfaction: Mediator of self-rated health (SRH)

**DOI:** 10.3389/fpsyt.2022.937685

**Published:** 2022-12-07

**Authors:** Weixi Kang

**Affiliations:** UK DRI Care Research and Technology Centre, Department of Brain Sciences, Imperial College London, London, United Kingdom

**Keywords:** smoke, smokers, smoking frequency, tobacco, tobacco use, cigarette, self-rated health, life satisfaction

## Abstract

**Background:**

It is well-established that smoking is associated with life satisfaction. However, much less is known about how smoking frequency is related to life satisfaction and if self-rated health (SRH) mediates such a relationship. This is important to understand because life satisfaction is related to a lot of outcomes such as morbidity and mortality. The aim of the current study is to test whether smoking frequency relates to life satisfaction *via* SRH pathway.

**Method:**

Data were extracted from Wave 7 (collected between 2015 and 2016), Understanding Society: the UK Household Longitudinal Study (UKHLS). After removing non-smokers and participants with missing variables of interest, 5, 519 smokers out of 39, 293 participants remained for further analysis. Correlation coefficients were calculated between smoking frequency, SRH, and life satisfaction. Mediation analysis was performed by taking smoking frequency as the predictor, SRH as the mediator, life satisfaction as the outcome variable, and demographics as covariates using the mediation toolbox on MATLAB 2018a with 10000 bootstrap sample significance testing (https://github.com/canlab/MediationToolbox).

**Results:**

The current study found a negative correlation between smoking frequency and life satisfaction [r = −0.09, 95% C.I (−0.12, −0.06), *p* < 0.001] and between smoking frequency and SRH [r = −0.17, 95% C.I (−0.14, −0.19), *p* < 0.001], and a positive correlation between SRH and life satisfaction [r = 0.44, 95% C.I (0.41, 0.46), *p* < 0.001]. Results from the mediation analysis revealed that there is a significant effect of Path a [i.e., smoking frequency to SRH; β = −0.02, *p* < 0.001, 95% C.I. (−0.02, −0.02)], Path b [SRH to life satisfaction; β = 0.68, *p* < 0.001, 95% C.I. (0.66, 0.69)], Path c' [direct effect; β = −0.01, *p* < 0.01, 95% C.I. (0.66, 0.69)], Path c [total effect; β =-0.02, *p* < 0.001, 95% C.I. (−0.02, −0.02)], and Path a^*^b [mediation effect; β = −0.01, *p* < 0.001, 95% C.I. (−0.01, −0.014)].

**Conclusion:**

SRH partially mediated the negative relationship between smoking frequency and life satisfaction. Findings from the current study may imply that antismoking campaigns and pamphlets are needed to counter the promotion of smoking by the tobacco industry. Moreover, interventions are needed for current smokers to reduce their smoking frequency to improve their life satisfaction, which can promote life satisfaction and positive outcomes associated with better life satisfaction.

## Introduction

According to the newest report released by the Office for National Statistics, there were around 5.5 million (13.5%) adults who smoked cigarettes in the first quarter of 2020 in the United Kingdom. Moreover, there were 4.9 million people who smoked cigarettes in Quarters 2 to 4 in the United Kingdom (1). It is well-acknowledged that smoking brings both negative physical and health consequences [e.g., (2)]. Moreover, long-term health risks of smoking include the risk of diseases such as lung cancer, cancer of the upper aerodigestive areas, bladder cancer, stroke, emphysema, heart attack, and various other conditions (3). Although the physical health consequences of smoking are well-established, much less is known about how smoking frequency could negatively impact psychological wellbeing such as life satisfaction given life satisfaction is closely related to morbidity (4) and mortality (5).

Life satisfaction is defined as the degree that a person likes or dislikes his or her life (6), which measures life to which extent a person finds a life of high quality. In the literature, the terms life satisfaction, subjective wellbeing, and happiness are often used interchangeably. Although this may not be correct, it makes a lot of sense given these terms overlap to a certain degree (7). The construct happiness plays a quite important role in people's life across the globe as evidenced by ongoing projects such as “The World Happiness Report” (http://worldhappiness.report/), which demonstrated that happiness is pursued by almost everyone (8). However, happiness can be hard to frame (9). While happiness is the overarching term being used, well-being may be understood as a more distinct part of happiness, thus making it better to be framed and analyzed (7). Moreover, there are two components of subjective wellbeing including the affective and cognitive components (10). Specifically, positive and negative effects are emotional aspects of subjective wellbeing whereas life satisfaction is the cognitive aspect of subjective wellbeing (11).

There are a few studies that have looked at the relationship between smoking and life satisfaction. For example, Zullig et al. (12) found that smoking cigarettes, chewing tobacco, marijuana, cocaine, and uses of alcohol, steroids, and drugs were associated with black and white people. Moreover, the age of the first cigarette, alcohol, marijuana, and cocaine use was significantly associated with reduced life satisfaction (12). Piko et al. (13) surveyed high school students aged 13–20 in urban and metropolitan areas from Ames, Iowa (USA), Szeged (Hungary), Izmir (Turkey), and Lublin and Warsaw (Poland). Piko et al. (13) found that high life satisfaction is associated with lower smoking rates across countries. More recently, Heshmat et al. (14) investigated how life satisfaction is related to both active and passive smoking in 1480 school students selected from both urban and rural areas across 30 provinces of Iran. Heshmat et al. (14) found life satisfaction is negatively affected by passive and active smoking in children and adolescents. Similarly, Barros (15) found reduced life satisfaction in smoking adults compared to non-smoking adults. Furthermore, Bogart et al. (16) investigated how cigarette, alcohol, marijuana, and hard drug use in adolescents could predict life satisfaction when they grew up into young adults. Results from their multivariate models suggested that the use of cigarettes at age 18 was associated with lower life satisfaction scores at age 29 after controlling for behavioral, environmental, and social factors. Similar results were found by research on the consequences of hookah use in American adults [e.g., (17)]. In particular, hookah users have considerably lower levels of ideal subjective wellbeing than non-hookah users (17). More recently, Xie et al. (18) found that smokers are more likely to have low life satisfaction. Self-rated health (SRH) is a single-item measurement that tries to assess health made up of biological, mental, social, and functional elements. Moreover, it has been suggested that poor SRH has been consistently found to be able to predict cardiovascular disease [e.g., (19)] and mortality [e.g., (20)] across several populations. There are some studies that have investigated the relationship between smoking and SRH. For instance, two earlier studies have identified that teenagers who smoked regularly had a higher chance of having poor SRH (21, 22). In addition, both active and passive smoking is negatively associated with SRH in Iran teenagers (14) but occasional smoking had no impact on SRH (23). In Chinese people, smoking is also negatively associated with SRH in both teenagers (24) and adults (25). However, it remains unclear how smoking frequency is related to life satisfaction and SRH.

Moreover, there are strong positive links between life satisfaction and SRH across countries and age groups [e.g., (26, 27)]. Indeed, good health is obviously one of the most important things to people and is often used as a source for people to evaluate overall life satisfaction (26). Thus, it is reasonable to speculate that smoking frequency negatively relates to life satisfaction through SRH pathway. Thus, the aim of the current study is to test if the smoking frequency is related to life satisfaction *via* SRH pathway.

## Methods

### Data

The current study extracted data from Wave 7, Understanding Society: the UK Household Longitudinal Study (UKHLS), which has been collecting annual information from the original sample of UK households since 1991 [when it was previously known as The British Household Panel Study (BHPS)]. Data in Wave 7 was collected between 2015 and 2016 (University of Essex). There were 5, 519 smokers out of 39, 293 participants in Wave 7. After removing missing variables of interest, 4, 944 participants with a mean age of 43.57 (S.D. = 15.98) were left for further analysis (Table 1). On average, they smoked 11.74 (S.D. = 7.78) cigarettes per day and had a mean SRH score of 3.06 (S.D. = 1.08) and life satisfaction score of 4.82 (S.D. = 1.62).

**Table 1:** Descriptive statistics with information about age, sex, monthly income, education, marital status, residence, the number of cigarettes smoked, SRH, and life satisfaction.

	**Mean (range)**	**S.D**.
Age	43.57(16-95)	15.98
Monthly income	1,334.84(0–23,929.17)	1,031.69
Number of cigarettes smoked per day	11.74 (0-70)	7.78
SRH	3.06(1-5)	1.08
Life satisfaction	4.82(1-7)	1.62
	***N***	**%**
**Sex**		
Male	2,434	49.23
Female	2,510	50.77
**Education**		
College	954	19.30
Below college	3,990	80.70
**Marital status**		
Single	2,291	46.34
Married	1,657	33.52
Divorced/separated/widowed	996	20.15
**Residence**		
Urban	3,990	80.70
Rural	954	19.30

### Measures

#### Smoking frequency

Smoking frequency was measured by letting participants answer the question: “Approximately how many cigarettes a day do you usually smoke, including those you roll yourself?”

#### Life satisfaction

Participants answered the question “How dissatisfied or satisfied are you with… your life overall?” using a 7-point scale ranging from 1 (not satisfied at all) to 7 (completely satisfied). According to Lucas and Donnellan (28), the reliability of this single-item measurement is at least 0.67.

#### SRH

Participants answered the question, “In general, would you say your health is...” using a 5-point scale ranging from 1 (excellent) to 5 (very poor). The SRH scores were reversed coded 1 = very poor and 5 = excellent to make the interpretation of the results more intuitive.

#### Demographics variables

Demographic variables include age, sex, highest educational qualification, legal marital status, employment status, monthly income, and whether participants live in urban or rural areas.

### Analysis

To test whether SRH serves as a mediator for the relationship between smoking frequency and life satisfaction, a mediation was performed by using the mediation toolbox on MATLAB 2018a with 10000 bootstrap sample significance testing by taking smoking frequency as the predictor, SRH as the mediator, and life satisfaction as the outcome variable with demographics as covariates (https://github.com/canlab/MediationToolbox). Moreover, correlations were calculated between smoking frequency, SRH, and life satisfaction on MATLAB 2018a using MATLAB native function.

## Results

The current study found that there are significant correlations between smoking frequency, SRH, and life satisfaction (Table 2). Specifically, there was a negative correlation between smoking frequency and life satisfaction [r = −0.09, 95% C.I (−0.12, −0.06), *p* < 0.001] and between smoking frequency and SRH [r = −0.17, 95% C.I (−0.14, −0.19), *p* < 0.001]. Finally, there was a positive correlation between SRH and life satisfaction [r = 0.44, 95% C.I (0.41, 0.46), *p* < 0.001].

**Table 2:** Correlations between smoking frequency, SRH, and life satisfaction.

	**Life satisfaction**	**SRH**
Smoking frequency	−0.09 [−0.12, −0.06]^***^	−0.17 [−0.14, −0.19]^***^
SRH	0.44 [0.41, 0.46]^***^	

^*^p < 0.05, ^**^p < 0.01,

*** *p* < 0.001.

**Figure 1:**
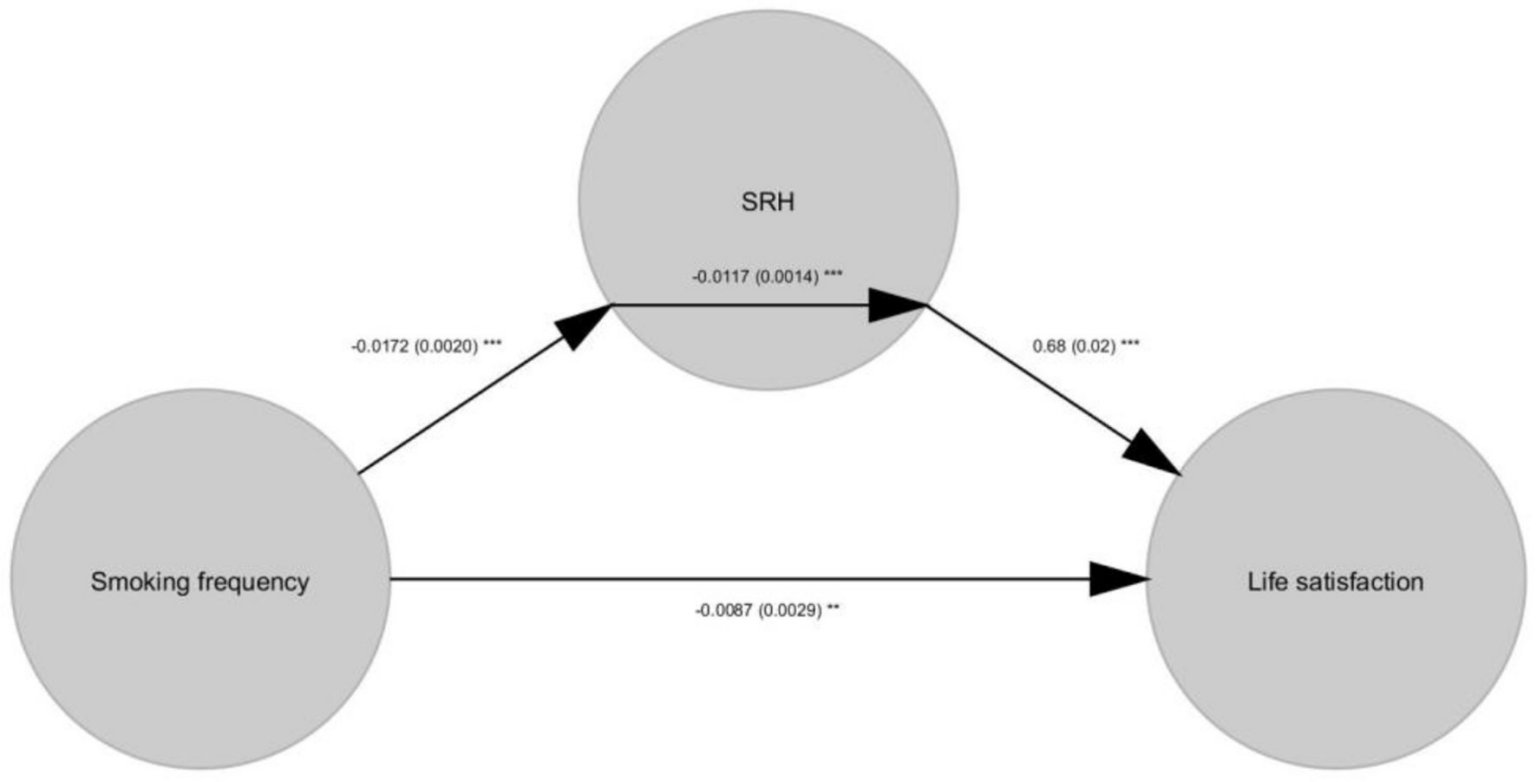
A path diagram that shows significant Path a (smoking frequency to SRH), Path b (SRH to life satisfaction), Path c (total effect), and Path a*b (mediation effect). The understandarize path coefficients are shown in the image with their standard errors. *p < 0.05, **p < 0.01, ***p < 0.001.

**Figure 2:**
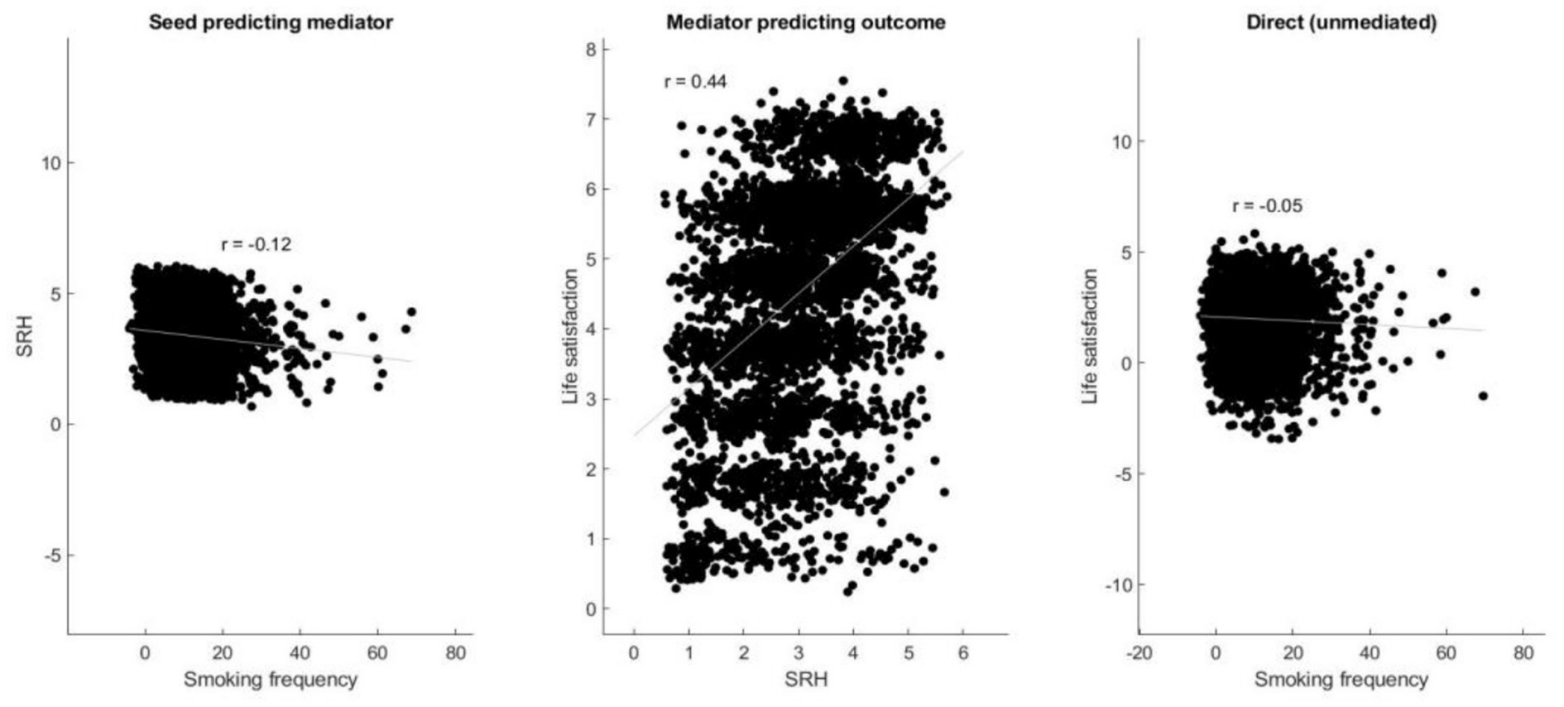
Scatter plots that show partial regression plots regarding smoking frequency predicting SRH, SRH predicting outcome, and unmediated direct effect.

Importantly, the current study found that there was a significant effect of Path a [i.e., smoking frequency to SRH; β = −0.02, *p* < 0.001, 95% C.I. (−0.02, −0.02)], Path b [SRH to life satisfaction; β = 0.68, *p* < 0.001, 95% C.I. (0.66, 0.69)], Path c' [direct effect; β = −0.01, *p* < 0.01, 95% C.I. (0.66, 0.69)], Path c [total effect; β = −0.02, *p* < 0.001, 95% C.I. (−0.02, −0.02)], and Path a^*^b [mediation effect; β = −0.01, *p* < 0.001, 95% C.I. (−0.01, −0.014); Table 3, Figures 1, 2].

**Table 3:** Coefficient, standard error, t value, Z score, 95% confidence interval, and associated *p*-value outputted from the mediation toolbox.

	**A**	**b**	**c'**	**c**	**ab**
Coeff	−0.02	0.68	−0.01	−0.02	−0.01
STE	0.00	0.02	0.00	0.00	0.00
t (~N)	−8.64	32.31	−2.97	−6.42	−8.32
Z	−3.73	3.67	−2.98	−3.70	−3.74
CI lb	−0.02	0.66	−0.01	−0.02	−0.01
CI ub	−0.02	0.69	−0.01	−0.02	−0.01
*p*	0.0002	0.0002	0.0029	0.0002	0.0002

## Discussion

Together, the current findings illustrated that smoking is negatively related to life satisfaction through SRH. To the best of my knowledge, the current study is the first one that identified the relationship between smoking frequency and life satisfaction and identified that SRH partially mediates such a relationship using a large nationally representative sample from the United Kingdom.

The finding that smoking frequency has a negative association with life satisfaction is largely consistent with previous studies [e.g., (12)]. Zullig et al. (12) found that life satisfaction was negatively affected by substance abuse behaviors including smoking cigarettes, chewing tobacco narijuanna, cocaine, and use of alcohol, steroids, and drug in adolescents. Piko et al. (13) investigated the relationship between adolescent smoking and social and personal consequences including life satisfaction across four different countries. Piko et al. (13) found lower life satisfaction was associated with adolescent smoking. They explain this effect as due to the self-concepts of individuals regarding their current and future status. In another word, the causality between these associations can be reciprocal and bidirectional, which means that one's perception of his or her situation could affect life satisfaction and smoking behavior (13).

There have been some studies that evaluated the associations between smoking and life satisfaction in adults. For instance, Barros et al. (15) compared life satisfaction in different smoking statuses among adults. Barros et al. (15) found that life satisfaction scores were higher in non-smokers. The associations between the use of other substances and life satisfaction have also been investigated. For instance, Grinberg (17) investigated the effects of hookah use on life satisfaction in American adults. Grinberg (17) found that subjective wellbeing was significantly lower in people who used hookah compared to non-hookah users.

In addition, the associations between smoking status in adolescence and life satisfaction in adulthood have been investigated. For instance, Bogart et al. (16) found that adolescents who used substances led to lower life satisfaction in early adulthood. Moreover, continued substance use may lead to lower life satisfaction compared to people who quit. Therefore, the current study quantified this association for the first time by demonstrating that the frequency of smoking is negatively associated with life satisfaction.

Consistent with previous studies, the current study also found that smoking frequency is negatively associated with SRH. Specifically, two previous studies identified that those who smoked daily had a higher chance of having poor SRH (21, 22). Wang et al. (24) studied SRH and smoking in Chinese adolescents and found a negative association between them. Moreover, Wang et al. (24) proposed that SRH could be used as a sensitive indicator of health in healthy adolescents who smoke. However, Vingilis et al. (23) investigated the role of occasional smoking and concluded that it had no significant effect on the SRH of adolescents.

Previous studies among adults have investigated the role of smoking and its impact on SRH and suggested that current smokers had poorer SRH than non-smokers and previous smokers in Hong Kong Chinese adults (25). However, there were also a few studies that reported no associations between smoking and SRH. For instance, Ramkumar et al. (29) compared the SRH in smokers and non-smokers in adults aged over 40 years old in Singapore. Ramkumar et al. (29) found no association between SRH and current smoking. However, the amount smoked was associated with poorer SRH.

Thus, the reason for the negative association between smoking and life satisfaction may pertain to the difference in the attitudes toward health behavior and health perception measures such as the SRH, which in turn affects life satisfaction. Indeed, this is what the current study exactly found. The strong positive link between SRH and life satisfaction is consistent with several previous studies across countries and age groups [e.g., (26, 27)]. Indeed, good health or subjective perceived good health certainly contributes to one's overall life satisfaction as health is one of the factors that make up overall life satisfaction (30). Thus, smoking directly affects SRH, which in turn results in decreased life satisfaction. However, this mediating relationship is only partial, meaning that smoking frequency may still affect life satisfaction directly.

Taken together, this study is the first study that investigated how smoking frequency is related to life satisfaction and demonstrated that SRH mediates the relationship between them. Despite its strength including large sample size and well-controlled demographic covariates, there are some limitations. First, this study is cross-sectional, which makes it hard to identify causal effects. Future studies should use a longitudinal design to study the established relationship in the current study. Second, all the measurements are self-reported, which can cause bias. Future studies should use more objective measurements such as biological assays. Finally, the current is based on a sample from the United Kingdom, which makes it hard to generate the current findings in other countries and contexts. Findings from the current study may imply that antismoking campaigns and pamphlets are needed to counter the promotion of smoking by the tobacco industry (24). Moreover, interventions are needed for current smokers to reduce their smoking frequency to improve their life satisfaction (31), which also can promote positive outcomes associated with better life satisfaction.

## Data availability statement

Publicly available datasets were analyzed in this study. This data can be found at: https://www.understandingsociety.ac.uk.

## Ethics statement

The studies involving human participants were reviewed and approved by University of Essex. The patients/participants provided their written informed consent to participate in this study.

## Author contributions

The author confirms being the sole contributor of this work and has approved it for publication.
